# A Comparison of Bottom-Up Proteomic Sample Preparation Methods for the Human Parasite *Trichomonas vaginalis*

**DOI:** 10.1021/acsomega.3c10040

**Published:** 2024-02-13

**Authors:** Anna-Lena Mayr, Karin Hummel, David Leitsch, Ebrahim Razzazi-Fazeli

**Affiliations:** † VetCore Facility, University of Veterinary Medicine, Veterinärplatz 1, 1210 Vienna, Austria; ‡ ISPTM, Medical University of Vienna, Kinderspitalgasse 15, 1090 Vienna, Austria

## Abstract

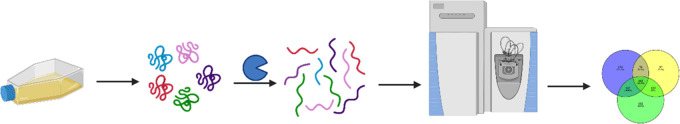

Bottom-up proteomic approaches depend on the efficient digestion of proteins into peptides for mass spectrometric analysis. Sample preparation strategies, based on magnetic beads, filter-aided systems, or in-solution digests, are commonly used for proteomic analysis. Time-intensive methods like filter-aided sample preparation (FASP) have led to the development of new, more time-efficient filter-based strategies like suspension trappings (S-Traps) or magnetic bead-based strategies like SP3. S-Traps have been reported as an alternative proteomic sample preparation method as they allow for high sodium dodecyl sulfate (SDS) concentrations to be present in the sample. In this study, we compare the efficiency of different protocols for FASP, SP3, and S-Trap-based digestion of proteins after extraction from *Trichomonas vaginalis*. Overall, we found a high number of protein IDs for all tested methods and a high degree of reproducibility within each method type. However, FASP with a 3 kDa cutoff filter unit outperformed the other methods analyzed, referring to the number of protein IDs. This is the first work providing the direct comparison of four different bottom-up proteomic approaches regarding the most efficient proteomic sample preparation protocol for the human parasite *T. vaginalis*.

## Introduction

Advances in mass spectrometry-based proteomics have enabled routine analysis of complex protein mixtures, which has led to the technology becoming a critical tool for the investigation of biological systems.^1−3^ By coupling ultraperformance liquid chromatography (UPLC) or nano-high-performance liquid chromatography (nano-HPLC) to a mass spectrometer, thousands of proteins can be identified in one run in samples of interest.^4−6^ Shotgun or bottom-up proteomics describes the enzymatic digestion of proteins to peptides prior to liquid chromatography-mass spectrometry (LC-MS) separation and measurement; thus, efficient digestion is necessary for the success of the experiment.^7,8^ Since the depth of coverage of the proteome is largely dependent on the sample preparation method chosen, identifying the optimal technique is crucial. These techniques, however, can vary greatly depending on the sample type, digestion^7−9^ (enzyme and conditions used), and lysis conditions,^10,11^ with each method having a set of advantages and disadvantages further described here.

Most commonly, detergents like sodium dodecyl sulfate (SDS) or chaotropic agents like urea are used to solubilize proteins in biological matrices. However, the removal of these substances is crucial prior to mass spectrometric analysis due to their ability to contaminate LC-MS systems and overshadow mass spectra.^12^ First introduced by Wiśniewski et al.,^13^ filter-aided sample preparation (FASP) has become a widely and frequently used processing technique for proteomic sample preparation in recent years.^1,14^ Solubilized protein samples are applied onto an ultrafiltration unit and are reduced and alkylated, followed by washing steps with a buffer, on-filter digestion, and elution of digested peptides. This method has been widely successful for a broad range of applications; however, the tedious, time-consuming nature of this protocol has led to the development of new strategies.^13,15^

Recently, Hughes et al. introduced single-pot, solid-phase-enhanced sample preparation (SP3) for proteomics.^16^ This method is based on carboxylate-functionalized paramagnetic beads with different hydrophilicities capturing proteins. Contaminations are removed by washing with ethanol and acetonitrile on a magnetic rack. This method provides an unbiased rapid and efficient proteomic workflow, with a high-throughput manner and large compatibility of different chemicals (e.g., detergents, chaotropic agents, salts). However, the range of protein amount that can be digested is limited, as a high protein content will lead to aggregation of beads causing stickiness and potential sample loss.^16−19^

Another method that is becoming more frequently used is a suspension trapping (S-Trap) method first proposed by Zougman et al.^20^ Here, proteins are lysed in 5% SDS. Phosphoric acid and a methanolic buffer solution are added to create a fine particulate suspension. The suspension is trapped on the filter matrix, SDS is removed by washing, and subsequently an on-filter digest is carried out with a protease of choice (e.g., trypsin) before LC-MS analysis. S-Trap reduces the hands-on time compared to the other methods tested while still providing the same advantages.^20,21^

In this study, we investigated various proteomic sample preparation strategies for the human parasite *Trichomonas vaginalis* in order to determine the optimal method. *T. vaginalis* is an anaerobic parasitic protist of the Excavate group, causing urogenital tract infections (trichomoniasis), mainly in women.^22,23^ It is one of the most frequent sexually transmitted pathogens worldwide, estimated by the WHO in 2016 to be responsible for approximately 156 million infections annually.^24^ Furthermore, it has a long list of serious associated complications, especially in women, including possible adverse pregnancy outcomes, infertility, and an increased risk of contracting human immunodeficiency virus (HIV).^24,25^ The parasite can be treated with the antibiotic metronidazole; however, the number of resistant strains has increased.^26^

*T. vaginalis* lysates were digested using two different FASP methods with various molecular weight cutoff filters, an S-Trap method, and an SP3 approach based on magnetic beads. After peptide analysis by nano-HPLC-MS/MS, protein IDs were compared for each sample preparation method in a qualitative approach, with special emphasis on the proteins with a molecular weight of around 10 kDa since these are assumed to play an important role in the formation of metronidazole resistance in this organism.^27^ Thus, a comparison of these sample preparation methods is of major importance to assess the suitability and robustness of *T. vaginalis* proteome analysis.

## Materials and Methods

### Reagents

Tryptic peptone, yeast extract, potassium dihydrogen phosphate, trichloroacetic acid (TCA), acetone, phosphate-buffered saline (PBS), and LC-MS grade acetonitrile and methanol were purchased from Merck (Darmstadt, Germany). Formic acid (FA) and trifluoroacetic acid (TFA) were purchased from Fisher Scientific (Schwerte, Germany). Maltose, iodoacetamide (IAA), triethylammonium bicarbonate buffer (TEAB), chloroacetamide (CAA), and dithiothreitol (DTT) were purchased from Sigma-Aldrich (Burlington, MA). Tris(2-carboxyethyl)-phosphine (TCEP), tris(hydroxymethyl)aminomethane (tris), sodium dodecyl sulfate (SDS), urea and thiourea, and 3-[(3-cholamidopropyl)dimethylammonio]-1-propanesulfonate (CHAPS) were purchased from Carl Roth (Karlsruhe, Germany). Isopropanol was bought from Honeywell (Morristown, NJ). Trypsin-LysC-Mix from Promega (Madison, WI) was used for digestion.

Suspension-traps were purchased from Protifi (Farmingdale, NY). Both types of FASP filters (3 and 10 kDa) were purchased from Pall Corporation (Port Washington, NY). Cytiva carboxylate-modified magnetic SpeedBeads were obtained from Sigma-Aldrich. Pierce C18 Spin Columns, Spin Tips, and Pierce 660 nm protein assay reagent were purchased from Thermo Scientific (Waltham, MA).

### Cell Culture and Protein Harvest

The *T. vaginalis* cell line TVC1 (ATCC 30001) was grown as previously described by Leitsch et al.^28^ Cells were harvested by placing flasks on ice, so cells would detach from the wall, followed by TCA/acetone precipitation. For this, cells were washed with 1 mL of PBS three times, and 500 μL of water and 1.5 mL of 13.3% TCA were added and incubated at −20 °C for 1 h. The cell pellet was then washed with 90% acetone five times, and the cell pellet was air-dried and dissolved in lysis buffer (7 M urea, 2 M thiourea, 4% CHAPS, 30 mM tris HCl, 1% DTT, at pH 8) at 25 °C and 700 rpm for 30 min. The cell pellet was then centrifuged (17,500*g*, 4 °C, 10 min), and the supernatant was stored at −80 °C for proteomic analysis. Total protein concentrations were determined using the Pierce 660 nm Protein Assay. Subsequently, 30 μg of protein was used for FASP, 20 μg of protein was used for S-Trap, and 10 μg of protein was used for SP3 digestion.

### Single-Pot Solid-Phase Sample Preparation (SP3)

For reduction and alkylation, 10 μg of protein, 200 mM of TCEP, and 800 mM of CAA in 100 mM of TEAB at an amount of 1:20 v/v were filled up with 100 mM of TEAB to a total volume of 28 μL, heated to 99 °C, and then incubated at 70 °C for 25 min. After cooling the sample on ice, magnetic beads, which were previously washed with LC-MS grade water, were added together with acetonitrile, and the mixture was sonicated for 10 min and incubated for binding at 25 °C and 550 rpm for 20 min. Then, beads were washed twice with 200 μL of 80% EtOH and once with 180 μL of acetonitrile. They were then air-dried and resuspended in 70 μL of 100 mM TEAB, followed by digestion with trypsin/LysC at a concentration of 1:25 (enzyme/protein w/w) overnight at 37 °C. Samples were then acidified with 40% of TFA to a final concentration of 1%, and peptides were desalted and cleaned using C18 Spin Tips. Before injection; samples were resuspended in 100 μL of 0.1% TFA.

### Filter-Aided Sample Preparation (FASP)

For both 3 and 10 kDa ultrafiltration units, 30 μg of protein was reduced with 20 mM of DTT for 30 min at 37 °C and then alkylated with 60 mM of IAA for 30 min at 25 °C in the dark on the filter. After washing twice with 100 μL of 50 mM tris, trypsin/LysC in 50 mM tris was added to a final enzyme concentration of 1:25 (enzyme/protein w/w) and digested overnight at 37 °C. Peptides were then eluted three times with 50 μL of 50 mM tris and acidified with conc. TFA for a pH < 2. Peptides were desalted and cleaned using Pierce C18 Spin Columns, and the eluate was then evaporated to dryness in a vacuum centrifuge. Before injection, samples were resuspended in 300 μL of 0.1% TFA.

### Suspension Trapping (Protifi S-Trap)

*T. vaginalis* extracts containing 5% SDS and 20 μg of protein were reduced with 32 mM of DTT for 30 min at 37 °C followed by alkylation with 125 mM of IAA for 30 min at 30 °C in the dark. Twelve % phosphoric acid (VWR, Radnor, PA) was then added to a pH ≤ 1, and the sample was applied onto the filter with S-Trap buffer (90% MeOH, 100 mM TEAB). The trap column was then washed with 150 μL of S-Trap buffer 6 times and centrifuged in between every step at 1000*g*. Samples were digested with 0.5 μg of trypsin/LysC in 50 mM of TEAB overnight at 37 °C. The next day, peptides were eluted with 40 μL of 50 mM TEAB and 0.2% formic acid followed by 35 μL of 50% acetonitrile and 0.2% formic acid and evaporated to dryness in a vacuum centrifuge. After redissolving in 100 μL of 0.1% TFA, peptides were desalted and cleaned using Pierce C18 Spin Columns. Before injection, samples were resuspended in 200 μL of 0.1% TFA.

### C18 Spin Tips

Tips were first prepared by adding 20 μL of wetting solution (80% acetonitrile in LC-MS grade water and 0.1% TFA) followed by 20 μL of 0.1% TFA and centrifuging at 1000*g* between every step. Samples were loaded onto the tips for desalting and cleaning of the peptides. Subsequently, they were washed twice with 20 μL of 0.1% TFA and eluted twice with 20 μL of elution buffer (80% ACN, 0.1% TFA in LC-MS grade water). Finally, samples were evaporated to dryness in a vacuum centrifuge.

### C18 Spin Columns

Columns were first activated by adding 200 μL of activation solution (50% acetonitrile in LC-MS grade water) twice, centrifuging at 1500*g* between every step. Then, columns were equilibrated with 200 μL of equilibration solution (5% ACN, 0.5% TFA in LC-MS grade water) twice. Samples were loaded onto the columns for the desalting and cleaning of the peptides. Subsequently, they were washed twice with 200 μL of washing solution (5% ACN, 0.5% TFA in LC-MS grade water) and eluted twice with 20 μL of elution buffer (70% ACN, 0.1% TFA in LC-MS grade water). Finally, samples were evaporated to dryness in a vacuum centrifuge.

### Mass Spectrometry and Data Analysis

All samples were analyzed using a nano-HPLC ultimate 3000 RSLC system (Dionex) coupled to a high-resolution Q-Exactive HF Orbitrap mass spectrometer (Thermo). The LC system was equipped with a 5 mm Acclaim PepMap μ-precolumn (300 μm inner diameter, 5 μm particle size, 100 Å pore size) for sample preconcentration and desalting. For sample loading and desalting 2% ACN in ultrapure water with 0.05% TFA as a mobile phase was used with a flow rate of 5 μL/min. For separation of peptides, a 25 cm Acclaim PepMap C18 column (75 μm inner diameter, 2 μm particle size, 100 Å pore size) with a flow rate of 300 nL/min was used. Solvent A consisted of 0.1% FA in ultrapure water, while solvent B consisted of 80% ACN with 0.08% FA. The following gradient was used for all samples: 4% B for 0–7 min, 4–31% B from 7 to 67 min, 31–44% B from 67 to 72 min, 44–95% B from 72 to 72.1 min, 95% B until 77 min, and re-equilibration at 4% B from 78 to 90 min.

The ion source was operated in a positive ion mode at 1.9 kV, and the ion transfer tube was maintained at 275 °C. Full MS scans were acquired from 350 to 2000 *m*/*z* at a resolution of 60,000, with an automatic gain control (AGC) target of 3 × 10^6^ ions and a maximum injection time of 50 ms. MS^2^ scans were performed at a resolution of 15,000 with the intensity threshold at 4 × 10^3^ and a maximum injection time of 50 ms. The AGC target was set to 5 × 10^4^ ions. An isolation window of 1.6 *m*/*z* was used for fragmentation with a normalized collision energy of 28 and dynamic exclusion was set at 30 s. Ions with a charge of +1, +7, +8, and >+8 were excluded from fragmentation. All samples were injected into nano-HPLC in duplicate. Database search was performed with Proteome Discoverer Software 2.4.1.15 (Thermo) using the *Sequest HT* search engine. Trypsin was set as the digestion enzyme with a maximum of two missed cleavages. Carbamidomethylation was set as a fixed modification. Oxidation (M), deamidation (NQ), acetylation (Protein N-term), Met-loss (Protein N-term (M)), Met-loss + acetyl (Protein N-Term (M)), and Gln → pyro-Glu (Q) were set as variable modifications. The precursor mass tolerance was 10 ppm, and the fragment mass tolerance was 0.02 Da. Spectra were searched in the Uniprot Trichomonas vaginalis database (tx5722, 51,768 sequences, www.uniprot.org, downloaded on 20.09.2020) using the cRAP database to filter out common contaminants (www.thegpm.org/crap/). The “Minora feature detector” node was used with a minimum trace length of 5 and a maximum ΔRT of isotope pattern multiplets of 0.2 min for peak and feature detection. Furthermore, in feature to ID linking, the peptide-spectrum match (PSM) confidence was set to at least high. Target decoy analysis was performed by searching a reverse database with a strict false discovery rate (FDR) of 0.01 and a relaxed FDR of 0.05 at the protein and peptide level.

For intensity-based label-free quantification (LFQ), protein abundance raw values were generated using the Proteome Discoverer software, including normalization to total area sums. Subsequently, the analysis of variance (ANOVA) analysis was performed in R version 4.3.0 (R Core Team, 2023). Preceding data import into R, protein abundances of technical replicates were accumulated by the mean using Microsoft Excel (version 16.78). Additionally, proteins with one or two missing values within the triplicate analyses were excluded from the ANOVA test to keep “ON/OFF” proteins while maintaining high data quality. Data generated by the ANOVA in R Studio were further utilized for statistical analysis and graphical display of data. Proteins identified with more than two tryptic peptides, quantified with at least one unique peptide, and displaying a fold change higher/lower than ±2-fold with an adjusted *p*-value for controlling the false discovery rate according to Benjamini–Hochberg lower than 0.05 were considered as up-/downregulated.

## Results and Discussion

Bottom-up proteomics approaches heavily rely on the solubilization of proteins from a given biological specimen to digest these proteins into peptides and identify them via mass spectrometric analysis. Several approaches comparing different shotgun proteomic sample preparation methods done in the past were regarding other organisms than *T. vaginalis*.^1,7,10,29^ Detergents like SDS are often used for solubilization of hydrophilic and hydrophobic proteins.^30,31^ This is also supported by the addition of urea as a chaotropic agent for protein denaturation and breaking of noncovalent bonds.^31^ Removal of these compounds is needed before LC-MS analysis in order to avoid interference during chromatographic separation as well as contamination of the mass spec instrument.^12,32^ The aim of this study was to establish an optimal sample preparation method for the human parasite *T. vaginalis*.

### Protein and Peptide Identification

**Figure 1:**
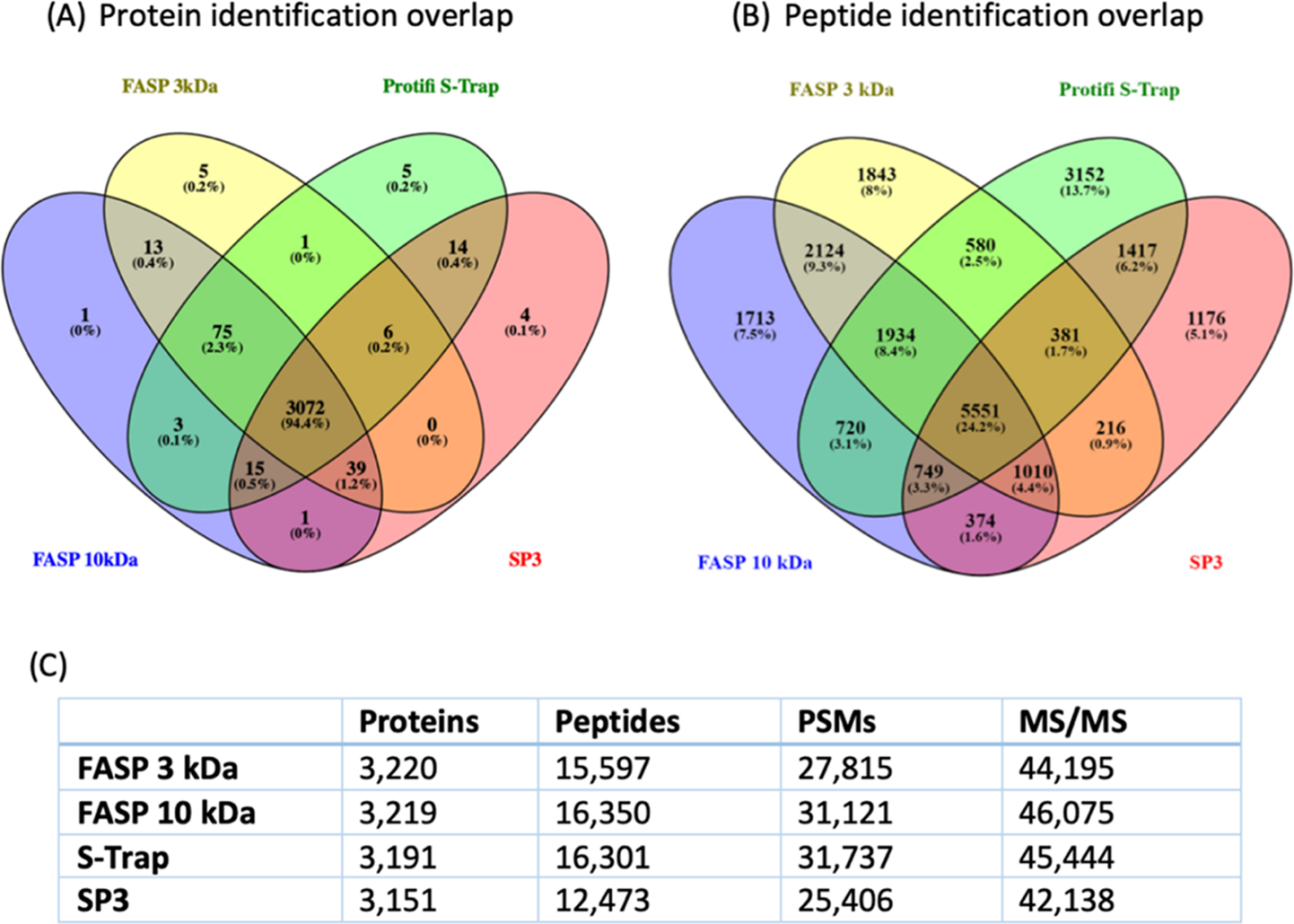
Protein and peptide identifications of tested sample preparation methods in three biological replicates with two technical replicates combined for each sample preparation method. (A) Overlap of identified protein groups. (B) Overlap of identified peptides. (C) Average number of identified protein groups, peptides, PSMs and MS/MS spectra.

In brief, two filter-based methods (filter-aided sample preparation, FASP, using a conventional molecular weight cutoff filter membrane with 3 and 10 kDa and Suspension-Traps employing a three-dimensional porous material as a filter medium) were compared to a method based on magnetic beads (single-pot solid-phase sample preparation, SP3). *T. vaginalis* lysate in lysis buffer, or lysis buffer additionally containing 5% SDS for S-Traps, was digested in three biological replicates for each method. Peptides were then separated using a reversed phase nano-HPLC column and analyzed with a high-resolution Q-Exactive HF mass spectrometer. Protein and peptide identification and quantification were performed using Proteome Discoverer software (Thermo). The overlap in identified proteins and peptides of all methods is plotted in Figure 1A,B, respectively. A table summarizing the number of identified protein groups, peptides, PSMs, and MS/MS spectra is shown in Figure 1C.

The total number of protein groups identified in each experiment ranged from 3151 to 3219 (Figure 1C). A major part of these protein groups (3072) was common in all four methods. The highest number of protein groups was identified by FASP 10 kDa with 3219, closely followed by FASP 3 kDa with 3211 protein groups identified. These also have the highest number of identified protein groups that overlap between these two methods, showing the high degree of similarity between these two methods, with only the filter cutoff size differing. For S-Trap, 3191 protein groups were found, while 3151 protein groups were identified with SP3. S-Trap and SP3 each show a minor number of proteins that were identified specifically in these methods. It shows that all presented sample preparation methods share a major part of identified proteins of *T. vaginalis*. This is indicated by an overlap in protein groups of 94.4% (Figure 1A). Especially with the two FASP filters of different kDa sizes, barely any difference in the proteins covered can be seen. Comparing the digestion methods to in-solution digest would have been interesting, as it has been suggested that filter-based and in-solution-based methods isolate different portions of the proteome.^1^ Since pre-experiments with in-solution digest in the applied lysis buffer composition resulted in low numbers of protein IDs and presence of CHAPS contamination during LC-MS analysis (found at *m*/*z* 614.9 (monomeric), *m*/*z* 1229.8 (dimeric)),^33,34^ this method was not further included into this study. All other protocols could remove CHAPS during sample preparation. Despite the manufacturer of S-Traps suggesting the use of at least 2% SDS, the same number of protein IDs was found when 5% SDS buffer was used as with our standard lysis buffer containing no SDS. Overall, there was a significant overlap of protein IDs in all methods (data not shown).

**Figure 2:**
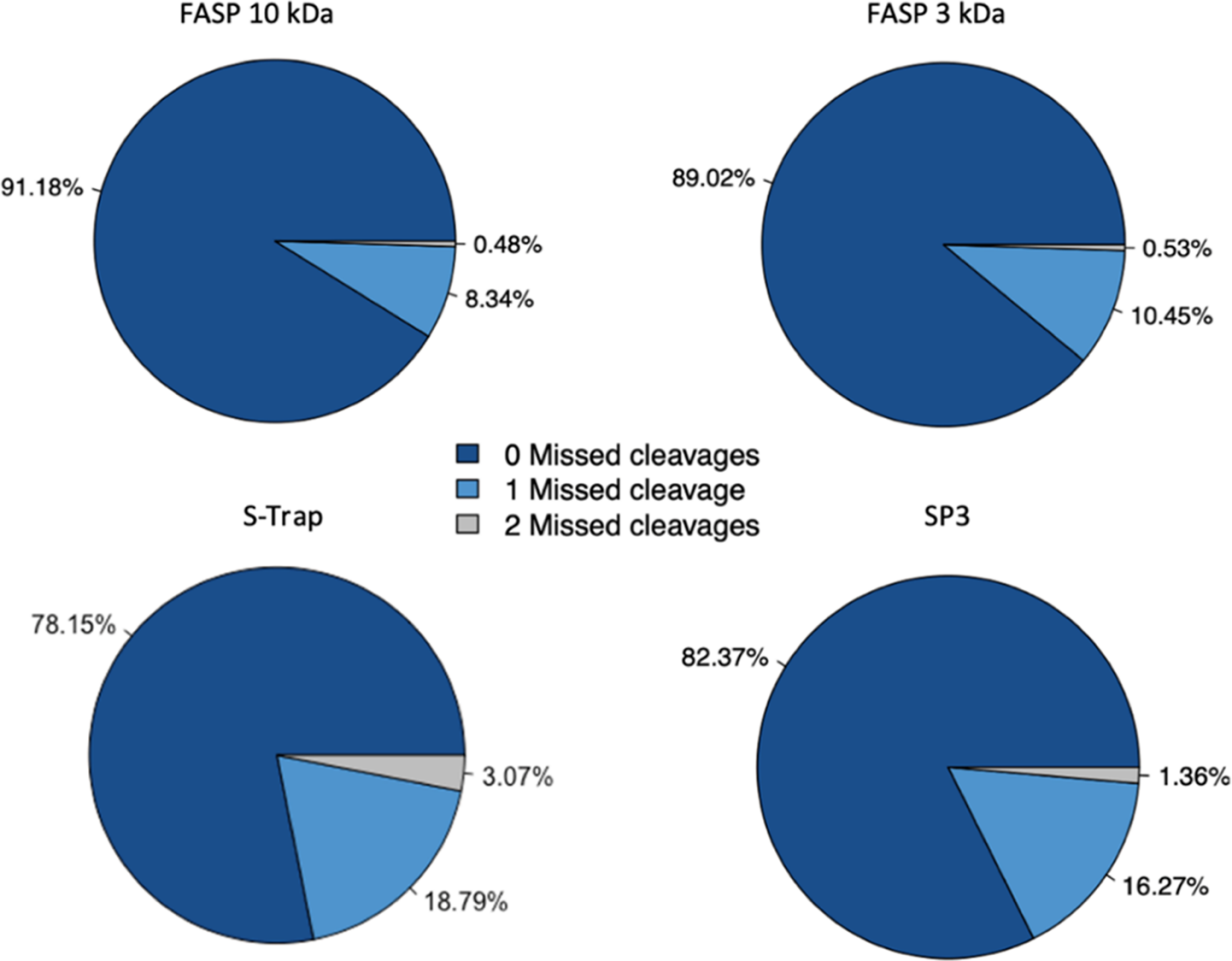
Trypsin efficiency was calculated across all experiments. The percentage of identified peptides containing either zero, one, or two missed cleavages is shown for each sample preparation method. Percentages shown are an average of three biological replicates with two technical replicates each.

When considering the number of peptides identified (Figure 1B), the total number ranged from 12,473 to 16,350 peptides. The three filter-based methods resulted in a larger number of peptides than SP3 based on magnetic beads. FASP 10 kDa resulted in the highest number of peptides identified with 16,350, followed by S-Trap with 16,301 peptides, FASP 3 kDa with 15,597 peptides, and SP3 with 12,473 peptides. It is remarkable that S-Trap has the second largest number of peptides identified, with only 49 peptides to FASP of 10 kDa having the highest number. When comparing this to the number of protein identifications, S-Trap has a slightly lower number than FASP 3 kDa. A reason could be the percentage of missed tryptic cleavages. For S-Trap, 21.9% of peptides contain at least one missed cleavage site (Figure 2), thus not resulting in the increased number of protein identifications one might suspect based on the high number of identified peptides.

The trend of missed cleavage sites was explored by investigating trypsin efficiency in all sample preparation methods. Both FASP methods showed superior trypsin efficiency, with around 90% of peptides identified having no missed cleavages. In comparison to that, with the S-Trap filters, only 78% of peptides identified had no missed cleavages. This is surprising, as the manufacturer states that the enclosure of trypsin together with the proteins in the pores of the S-Trap filters should lead to a rapid digestion, which should result in a lower number of missed cleavages.^35^ However, the enzymes need to move in order to encounter the protein and thus cleave it, which could be a possible explanation for the increased number of missed cleavages with S-Trap columns.^27,36^

An explanation for the good performance of FASP filters may be the high trypsin concentration present when samples are digested on a filter due to the proximity of trypsin and proteins on the filter. This trend in distribution was also observed in peptides with one and two missed cleavages. Furthermore, finding proteins with a small molecular weight like ferredoxins (10.7–13.9 kDa) and thioredoxin (12 kDa) is of special importance, as they are believed to play an important role in the formation of metronidazole resistance in *T. vaginalis*.^37^ Therefore, it is crucial to find a method able to digest these proteins efficiently. The molecular weight of identified proteins found was compared within the sample preparation methods (Supplemental Figure S1). This showed that FASP filters were superior in the digestion and identification of small proteins in comparison to S-Trap filters and SP3. However, there was barely a difference between FASP 10 kDa and FASP 3 kDa, going against the assumption, that the filter with the smaller cutoff size (FASP 3 kDa) would also result in a larger number of small proteins being identified.

In the present study, urea and thiourea were used in the lysis buffer. Still, lysis buffer containing SDS is often preferred for proteomic analysis due to its ability to extract protein with high efficiency.^10,30,31^ However, we found that FASP filters do not efficiently remove SDS, leading to contamination of the mass spectrometer (data not shown). In order to circumvent this, S-Trap filters are described in the literature to remove SDS.^1^ Also in the presented study, S-Trap columns were found to efficiently remove SDS, avoiding any interference in the LC-MS system. According to the manufacturer, Protifi concentrations of 2–15% SDS are required for effective column use;^28^ however, we achieved comparable results with a mix of urea and thiourea bypassing the use of SDS when extracting proteins from *T. vaginalis* (data not shown).

**Figure 3:**
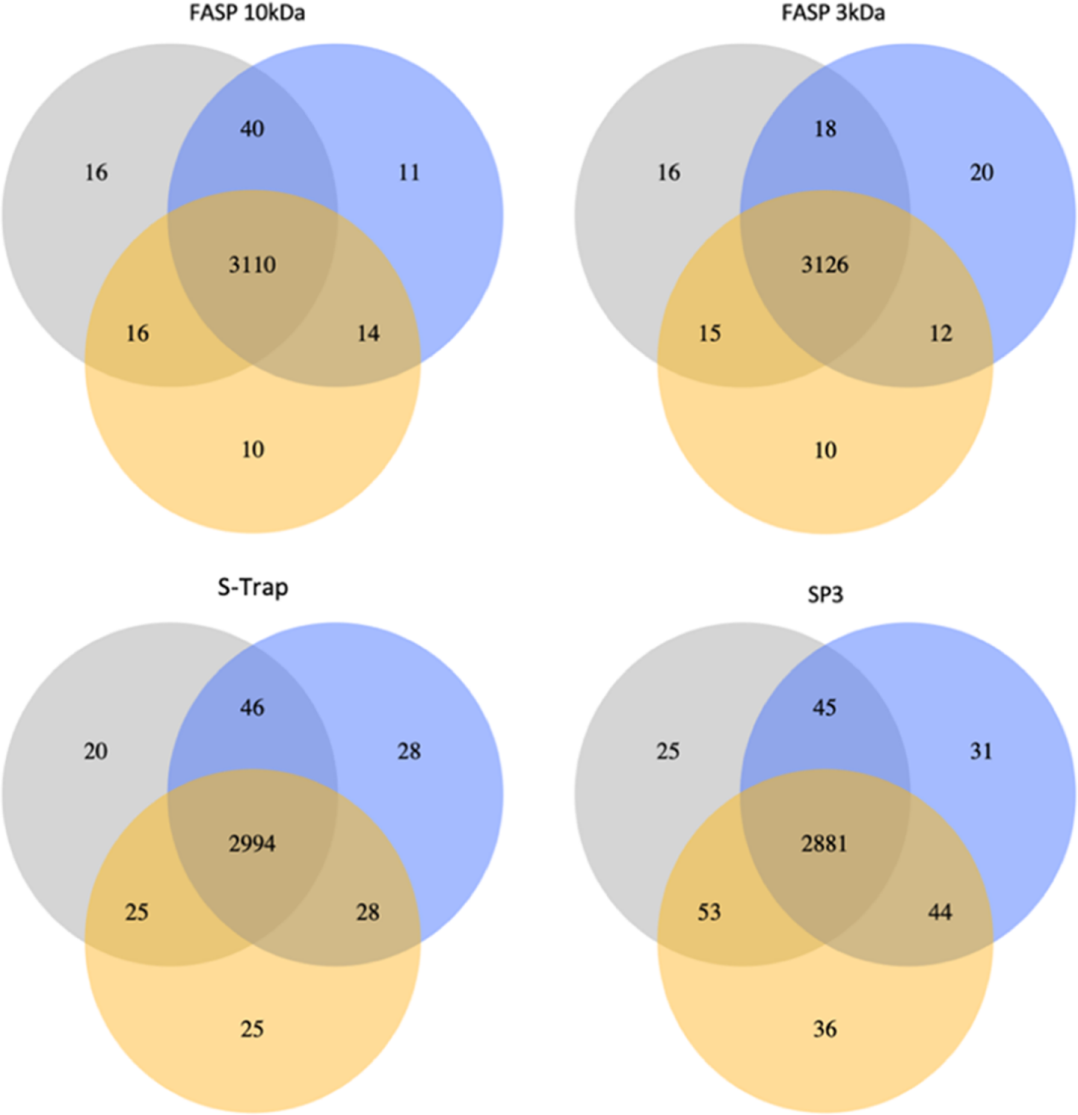
Three biological replicates on the protein level. Venn diagrams display an overlap in the identified protein groups as the average of two technical replicates for each digestion condition (FASP 10 kDa, FASP 3 kDa, S-Trap, SP3).

**Figure 4:**
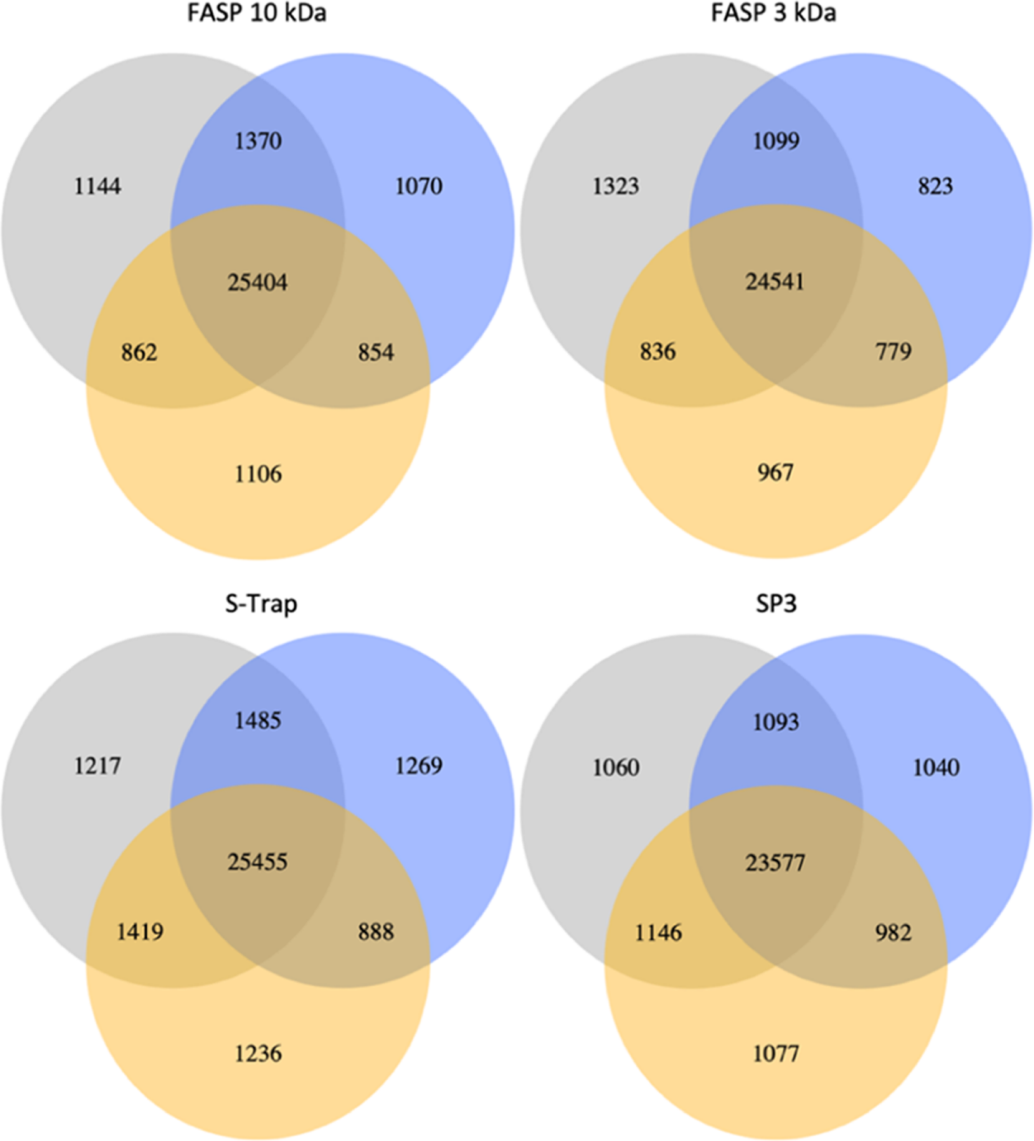
Three biological replicates on the peptide level. Venn diagrams display an overlap in the identified peptides as an average of two technical replicates for each digestion condition (FASP 10 kDa, FASP 3 kDa, S-Trap, SP3).

Reproducibility of all four digestion methods is shown in Figure 3. Three biological replicates were analyzed for each sample preparation method. The high overlap of proteins confirms good reproducibility for all four digestion methods, with FASP 3 kDa having the highest degree of overlap for all three biological replicates and SP3 having the lowest. The overlap of peptides for all sample preparation methods tested shows similar results; however, in this comparison, S-Trap has the lowest degree of overlap (Figure 4). Technical replicates for each biological sample of the protein digestion methods tested also showed a high degree of overlap (Supplemental Figure S2), verifying the stability of mass spectrometric analyses.

In general, the high degree of overlap for both proteins and peptides in all sample preparation methods tested across three biological replicates shows the high reproducibility of the methods used.

**Figure 5:**
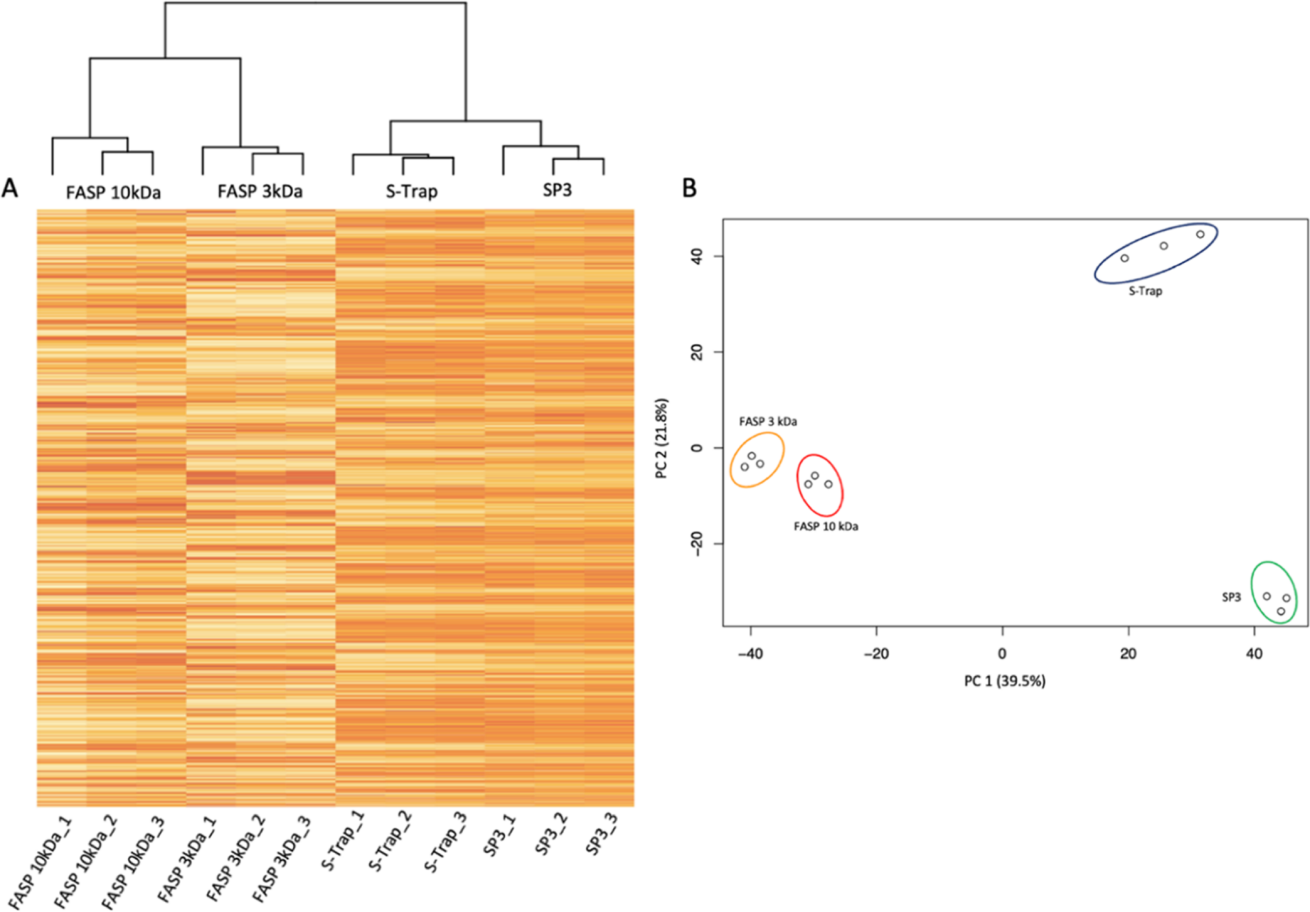
(A) Hierarchical clustering showing protein expression patterns of *T. vaginalis* for each sample preparation method. Red bands indicate higher protein expression for one method compared to the others, while the cream-colored bands indicate low protein expression compared to the other methods. (B) PCA score plot of FASP 10 kDa (red), FASP 3 kDa (yellow), S-Trap (blue), and SP3 (green).

Figure 5B shows the principal component analysis (PCA) of biological replicates of each sample preparation method being clustered together while clearly differentiated from the identical biological samples prepared with a different sample preparation method. FASP methods are found to be in proximity, unveiling the similarity of the part of the proteome covered by FASP despite the different filter cutoff sizes. It also shows that regardless of a few differences, the uncovered proteome is similar for each sample preparation method tested. This is also shown by the overlap of proteins and peptides identified (Figure 1A,B). Supplemental Figure 2 graphically displays the overlap of technical replicates, confirming the high reproducibility of the applied proteome analysis and instrument stability. Protein abundance values for all tested methods (FASP 10 kDa, FASP 3 kDa, S-Trap, and SP3) were used to perform unsupervised hierarchical clustering in R (Figure 5A). For all methods, the three biological replicates of each method tend to aggregate, with the FASP digests of different filter cutoff sizes grouping together, and SP3 and Protifi S-Trap clearly separated from FASP. This shows the similarity and degree of reproducibility between replicates while highlighting clear differences between FASP and both other methods.

**Figure 6:**
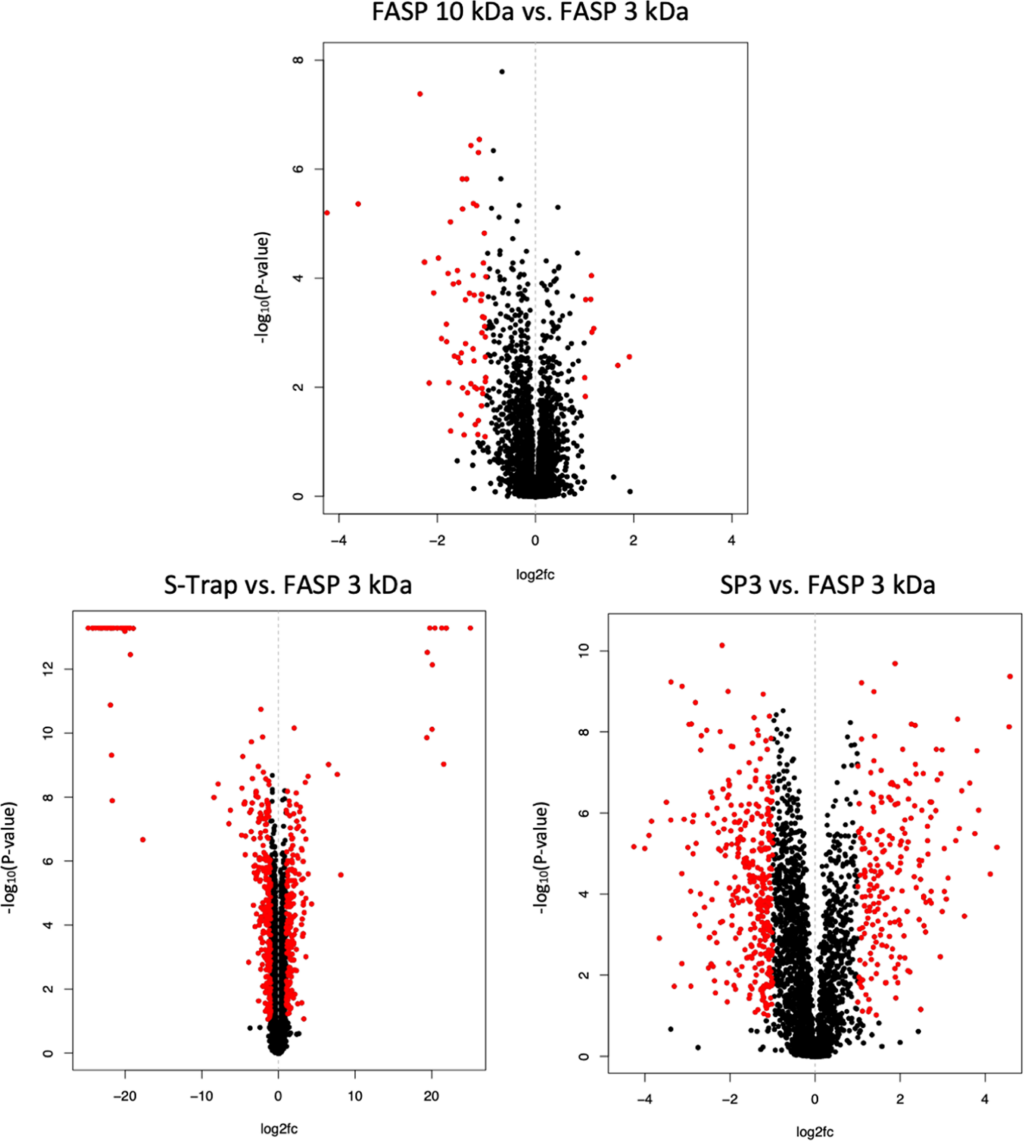
Volcano plots displaying the statistical *p*-value with the magnitude of abundance changes between each sample preparation method compared to FASP of 3 kDa. Red dots indicate proteins meeting the threshold for the significance of changes in protein abundance levels (log_2_ < 1 or log_2_ > 1 with a *p*-value of <0.05).

In addition, we quantified proteins in all experiments relative to FASP 3 kDa, creating volcano plots (Figure 6). FASP 3 kDa was chosen for comparison, as it was the best-performing method for analyzing the *T. vaginalis* proteome, as shown before. Red dots indicate proteins that met the threshold of differential regulation (log_2_ < 1 or log_2_ > 1 with an FDR-adjusted *p*-value of <0.05). As expected, both FASP methods identify a very similar subset of proteins, while Protifi S-Trap shows the highest deviation from FASP 3 kDa. Given the different conditions during digestion, it is expected that each method would favor a distinct part of the proteome. Still, this also shows the importance of quantitative studies needing to be identical and reproducible in their methodologies, as significant differences in the abundance of the peptides identified between methods are seen in Figure 6. Despite the similarity of the protein subsets identified by the different methods, proteins of specific interest were found to be enriched with FASP 3 kDa, thus rendering FASP 3 kDa as the most suitable sample preparation method for bottom-up proteomics regarding the human parasite *T. vaginalis*.

## Conclusions

This report describes in detail the similarities and differences of four proteomic sample preparation methods regarding the human parasite *T. vaginalis*. Proteins and peptides were identified using a bottom-up LC-MS/MS approach. FASP 3 and 10 kDa filters, S-Trap columns, and SP3 magnetic beads were compared. It was shown that FASP 3 kDa filters outperformed the other three methods, with the highest number of proteins and thus the largest section of the proteome covered. Enzymatic digestion using FASP 3 kDa filters identified 3220 protein groups over two technical and three biological replicates. Based on the percentage of no missed cleavages, trypsin performed with the highest efficiency of 91.2% in the FASP 3 kDa digest.

Each proteomic sample preparation method tested possesses several advantages and disadvantages. SP3 is cost-efficient and showed good reproducibility; however, it requires the most hands-on time out of all of the methods and yields the lowest number of proteins and peptides identified. S-Trap required a low hands-on time as well as good reproducibility with a high yield of identified proteins and peptides; however, it was the most expensive method tested.

Finally, we found that the FASP filters, especially the FASP 3 kDa filter, demonstrated the best overall performance, showing the highest number of proteins identified as well as good quantitative reproducibility and superior trypsin efficiency. Despite the overnight workflow and time-consuming centrifugation steps, it required little hands-on time and was placed in the midrange regarding costs. Overall, it outperformed the other methods with the total number of proteins identified, quantitative reproducibility, and ability to find proteins of a molecular weight of around 10 kDa, believed to be important for antibiotic resistance in *T. vaginalis*. The aim was to elucidate the optimal sample preparation method for the proteomic analysis of the human parasite *T. vaginalis*. FASP 3 kDa filters showed the best overall performance and thus proved to be the sample preparation method best suitable for the human parasite *T. vaginalis*.

## Supporting Information Available

(A) Analysis of weight distribution and (B) weight distribution for each sample preparation method (Figure S1); technical replicates of peptides (Figure S2) (PDF)
Protein abundance values (Table S1); script for ANOVA in R for *Trichomonas vaginalis* (Table S2); and ANOVA results in *Trichomonas vaginalis* (Table S3) (XLSX)
