# Supplementary material for: A Comparison of Bottom-Up Proteomic Sample Preparation Methods for the Human Parasite Trichomonas vaginalis
Source: ACS Omega. 2024 Feb 13;9(8):9782–91. doi: 10.1021/acsomega.3c10040 (PMC10905575; doi:10.1021/acsomega.3c10040)
Supplement: Supplementary file 1 — ao3c10040_si_001.pdf [file ao3c10040_si_001.pdf]

Supplemental information I

A comparison of bottom-up proteomic sample preparation methods for the human parasite *Trichomonas vaginalis*

Anna-Lena Mayr[a], Karin Hummel[a], David Leitsch[b], Ebrahim Razzazi-Fazeli[a]*

[a]VetCore Facility, University of Veterinary Medicine, Veterinärplatz 1, 1210 Vienna, Austria

[b]ISPTM, Medical University of Vienna, Kinderspitalgasse 15, 1090 Vienna, Austria

*Ebrahim.razzazi@vetmeduni.ac.at, +43 1250773216

Table of contents

Supplemental figure 1: (A) Analysis of weight distribution. Proteins that are below a molecular weight of either 10 kDa or 11 kDa cut-off are displayed for each method. (B) Weight distribution for each sample preparation method. You can see that the largest number of proteins identified, lies within a range of 10-30 kDa for all methods. SP3 also found a proportionally higher number of proteins in the range of 30-40 kDa in comparison to the other methods. This could be, because SP3 is the only method tested that is not filter based.

Supplemental figure 2: Technical replicates of peptides.

Supplemental information II: contained in separate excel file.

- Supplemental table 1: Protein abundance values
- Supplemental table 2: Script for ANOVA in R for *Trichomonas vaginalis*
- Supplemental table 3: ANOVA results for *Trichomonas vaginalis*

Supplemental Figure S1

(A)

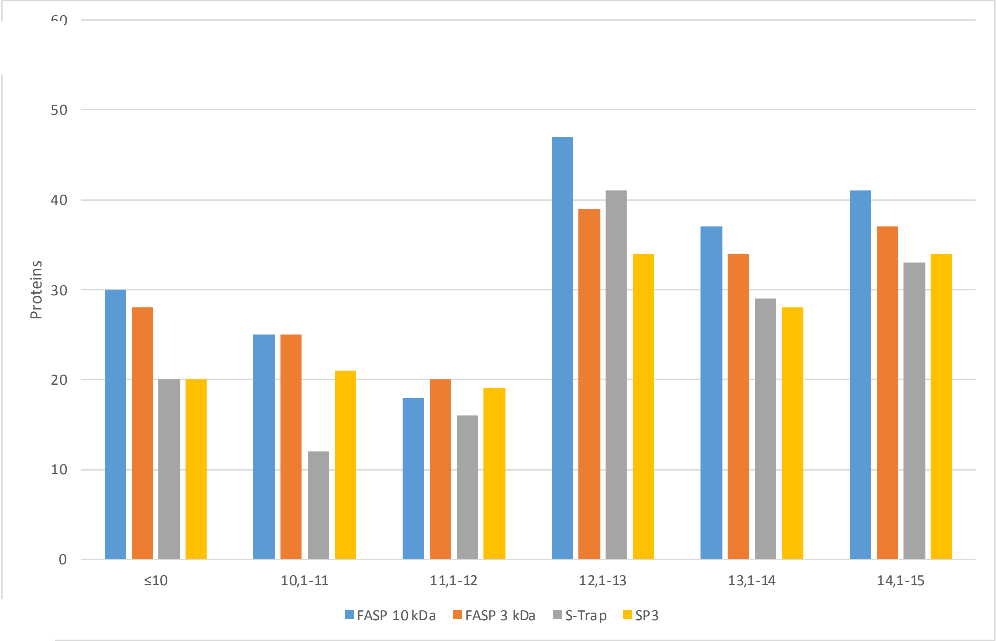

(B)

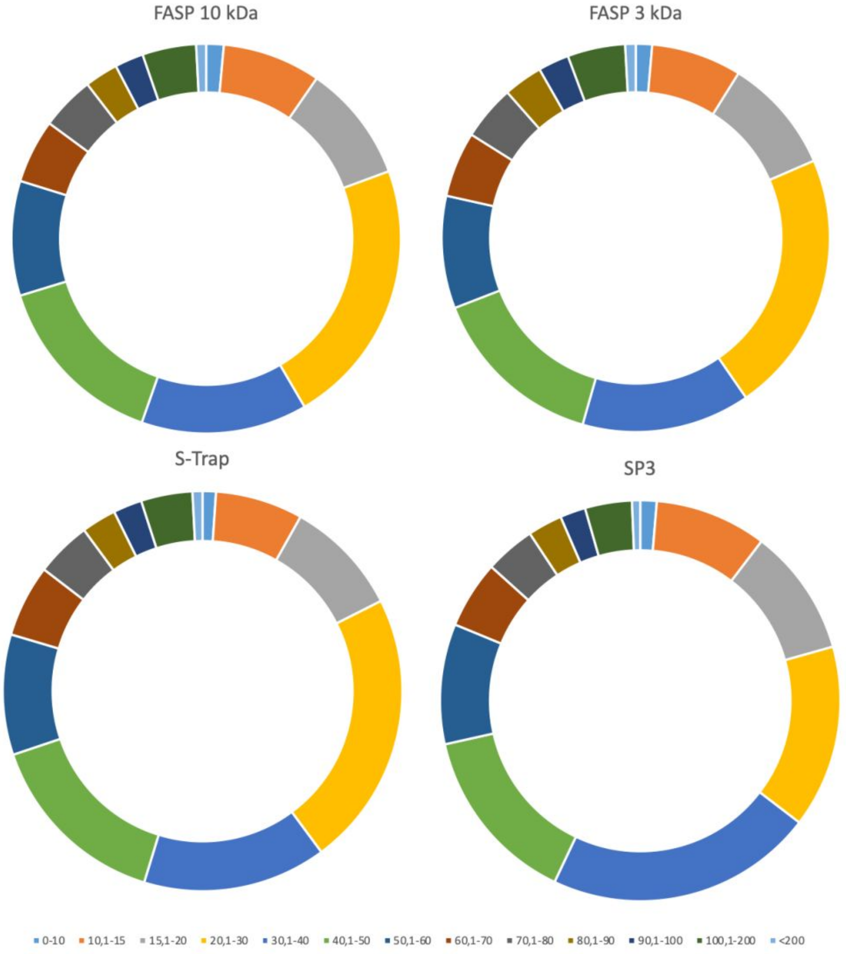

Supplemental Figure 1: (A) Analysis of weight distribution. Proteins that are below a molecular weight of either 10 kDa or 11 kDa cut-off are displayed for each method. (B) Weight distribution for each sample preparation method. You can see that the largest

number of proteins identified, lies within a range of 10-30 kDa for all methods. SP3 also found a proportionally higher number of proteins in the range of 30-40 kDa in comparison to the other methods. This could be, because SP3 is the only method tested that is not filter based.

Supplemental Figure S2

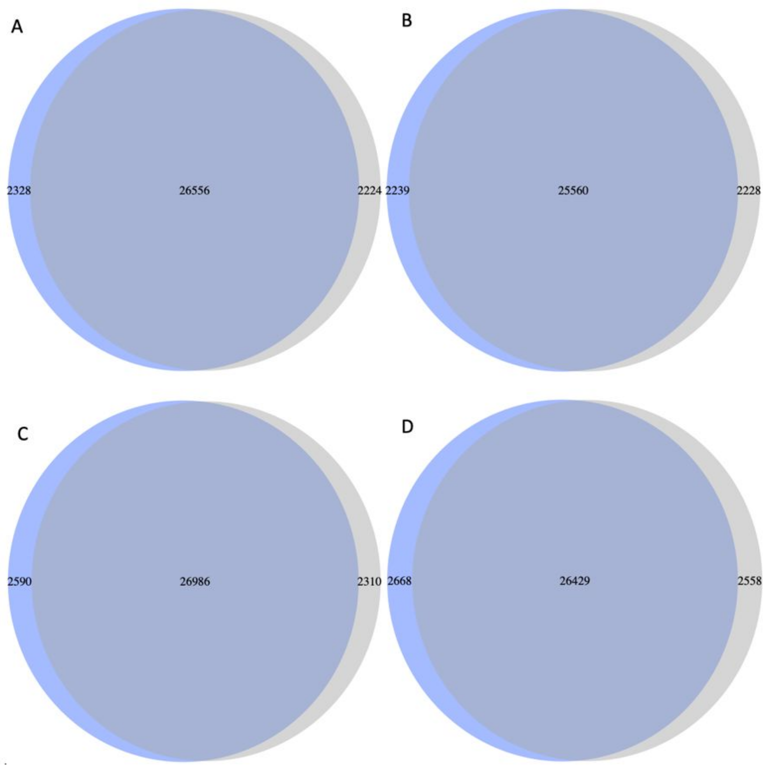

Supplemental Figure 2: Technical replicates of peptides. (Mean value for three biological replicates) (A) FASP 10 kDa, (B) FASP 3 kDa, (C) S-Trap, (D) SP3
